# No association between methotrexate and impaired bone mineral density in a cohort of patients with polymyalgia rheumatica, giant cell arteritis, granulomatosis with polyangiitis and other vasculitides—a cross-sectional analysis with dose–response analyses

**DOI:** 10.1007/s00296-023-05286-6

**Published:** 2023-02-22

**Authors:** Andriko Palmowski, Mitsuteru Akahoshi, Burkhard Muche, Zhivana Boyadzhieva, Sandra Hermann, Chikashi Terao, Edgar Wiebe, Frank Buttgereit

**Affiliations:** 1 Department of Rheumatology and Clinical Immunology, Charité - Universitätsmedizin Berlin, Corporate Member of Freie Universität Berlin and Humboldt-Universität zu Berlin, Berlin, Germany; 2 Department of Rheumatology, Faculty of Medicine, Saga University, Saga, Japan; 3 Laboratory for Statistical and Translational Genetics, RIKEN Center for Integrative Medical Sciences, Yokohama, 230-0045 Japan; 4 Section for Biostatistics and Evidence-Based Research, The Parker Institute, Bispebjerg and Frederiksberg Hospital, Frederiksberg, Denmark

**Keywords:** Vasculitis, Osteoporosis, Methotrexate, Bone mineral density

## Abstract

**Objective:**

To investigate whether methotrexate (MTX) use is associated with bone mineral density (BMD) in patients with polymyalgia rheumatica (PMR) and various forms of vasculitis.

**Methods:**

Rh-GIOP is a cohort study designed to evaluate bone health in patients with inflammatory rheumatic diseases. This cross-sectional analysis assessed the baseline visits of all patients with PMR or any kind of vasculitis. Following univariable analysis, multivariable linear regression analysis was performed. The lowest T-score of either the lumbar spine or the femur was chosen as the dependent variable to examine the relationship between MTX use and BMD. These analyses were adjusted for a variety of potential confounders, including age, sex, and glucocorticoid (GC) intake.

**Results:**

Of 198 patients with PMR or vasculitis, 10 patients were excluded for very high GC dose (*n* = 6) or short disease duration (*n* = 4). The remaining 188 patients had the following diseases: PMR 37.2%, giant cell arteritis 25.0%, granulomatosis with polyangiitis 16.5%, followed by rarer diseases. The mean age was 68.0 ± 11.1 years, mean disease duration was 5.58 ± 6.39 years, and 19.7% had osteoporosis by dual x-ray absorptiometry (T-score ≤ −2.5). 23.4% were taking MTX at baseline with a mean dose of 13.2 mg/week (median: 15 mg/week). 38.6% of those used a subcutaneous preparation. MTX users had similar BMD compared to non-users (minimum T-scores −1.70 (± 0.86) versus −1.75 (± 0.91), respectively; *p* = 0.75). There was no statistically significant dose–response relationship: neither current nor cumulative dose were associated with BMD in unadjusted or adjusted models (current dose: slope −0.02; −0.14 to 0.09; *p* = 0.69; cumulative dose: slope −0.12; −0.28 to 0.05; *p* = 0.15).

**Conclusion:**

In the Rh-GIOP cohort, MTX is used in about a quarter of patients with PMR or vasculitis. It is not associated with BMD levels.

## Introduction

Patients with polymyalgia rheumatica (PMR) and vasculitides are at an increased risk of osteoporosis (OP) and fragility fractures [1, 2]. The underlying causes have not been fully identified yet as recent studies challenge the old assumption that glucocorticoids (GCs) are the main culprits. In a recent analysis of the Rh-GIOP cohort, GCs were not associated with bone mineral density (BMD) in patients with PMR and vasculitides [3]. As it is well-known that inflammation increases the risk of fracture and deteriorates bone health, [4–7], we also assessed CRP but found no evidence for an association between CRP and bone density.

Methotrexate (MTX) is recommended as a disease-modifying anti-rheumatic drug (DMARD) in PMR [8] and vasculitides such as giant cell arteritis (GCA) or granulomatosis with polyangiitis (GPA) [9, 10]. While controlling inflammation with MTX might be beneficial for bone health, the occurrence of MTX-induced osteopathy has been described in patients with rheumatic diseases, [11–13] and high-dose MTX use (as in cancer treatment) was linked to bone loss [14]. In the study at hand, we assessed whether chronic low-dose MTX (as used to treat vasculitides) is linked with changes in BMD in patients with PMR and vasculitides.

## Methods

Rh-GIOP (Glucocorticoid-Induced Osteoporosis in Patients with chronic inflammatory Rheumatic diseases or Psoriasis) is an ongoing prospective, monocentric observational cohort study conducted at Charité–Universitaetsmedizin Berlin, a large tertiary care university hospital. It is registered with clinicaltrials.gov (https://clinicaltrials.gov/ct2/show/NCT02719314) and has received an ethics approval from the local ethics committee at Charité–Universitätsmedizin Berlin (EA1/367/14; Jan 27, 2015).

We include patients who (a) have a chronic inflammatory rheumatic disease or (for comparison) psoriasis, (b) are at least 18 years of age, and (c) have an indication for a bone density measurement as recommended by the “Dachverband Osteologie” (DVO) [15]. The DVO, representing a tri-national umbrella association of currently 21 medical and scientific professional societies which deal with bone diseases, has established guidelines for the care of patients with bone diseases, which we follow in our Rh-GIOP study [15]. Patients must be able to provide informed consent. Breastfeeding, lactating, and pregnant women are not included.

At each visit, a multitude of clinical variables are noted—details have been recently published [16]. These include demographics such as age, sex, and body mass index (BMI); variables concerning GC use such as current dose, cumulative dose, and duration of GC therapy; variables describing the underlying rheumatic disease including disease duration, disability, and anti-rheumatic medication, variables linked to bone health such as family history of osteoporosis, prior fractures, and anti-osteoporotic treatment. Both general and bone-specific laboratory tests are performed.

BMD is measured by dual X-ray absorptiometry (DXA) using a Lunar Prodigy scanner (GE Medical Systems Lunar Corporation, Madison, Wisconsin, USA) using the manufacturer’s reference database. The results are presented as T-scores. According to the World Health Organization, T-scores of ≥ −1.0 are considered normal, < −1 to > −2.5 osteopenic, and ≤ −2.5 osteoporotic [17]. A trained operator did the measurements. Quality control measures of the device are performed as recommended by the manufacturer.

For the analysis at hand, we looked at baseline data of patients with PMR or any type of vasculitis. Patients with GC doses ≥ 100 mg prednisone equivalent per day were excluded to avoid distortion of results due to extreme outliers (confirmed by Rosner’s test [18] as were patients with short disease duration < 3 months) because no significant effect of any drug on BMD is expected in the short term. No formal sample size calculations were performed; rather, we included all enrolled and eligible patients.

Descriptive analyses were performed, with values displayed as mean/standard deviation (SD) for normally distributed continuous variables and median/interquartile range (IQR) for non-normally distributed ones. For inferential statistics, *t* tests and univariable linear regression analyses were performed first, then followed by multiple linear regression analyses (to adjust for confounding variables). Preselected potential confounders included in multiple regression models were the following: age, sex, BMI, current GC dose, cumulative GC dose, disease duration, prior vertebral fracture, prior non-vertebral fracture, proton pump inhibitors, 25-(OH)-Vitamin D levels, diabetes mellitus type II, alcohol consumption, and frequency of physical exercise. A dose–response analysis was conducted within the group of MTX users by including current and cumulative MTX doses as independent continuous variables. As this study might suffer from confounding by indication, i.e., patients with more severe disease might be more likely to have osteoporosis and be more likely to receive MTX, a sensitivity analysis including serum C-reactive protein levels (as a measure of inflammation and a surrogate measure for disease activity) was conducted. For the analyses pertaining to cumulative MTX doses, extreme outliers detected with Rosner’s test were excluded (i.e., cumulative dose > 15 g) [18]. No outliers were detected for current MTX dose.

*P* values lower than 0.05 were considered statistically significant. All statistical analyses were performed using JMP Pro, version 16.0 (SAS Institute Inc., Cary, NC, USA), SPSS (IBM Corp., Armonk, NY, USA), and R (R Foundation for Statistical Computing, Vienna, Austria).

## Results

### Baseline characteristics

We included 198 patients. Of these, ten patients were excluded due to very high GC dose (> 100 mg/day, *n* = 6) or short disease duration (< 3 months, *n* = 4). Of the remaining 188 patients, 70 (37%) had PMR, 47 (25%) had GCA, and 10 (16%) had GPA. The overall prevalence of osteoporosis measured by DXA (T-scores ≤ -2.5) was 19.7%. Sixty-two patients (33.0%) had a history of fractures due to inadequate trauma. Forty-four (23.4%) patients were treated with MTX (mean dose of 13.2 mg/week), of which 59.1% were administered orally—38.6% were administered subcutaneously (missing data for one patient corresponding to 2.3% of the MTX user sample). In our sample, 45.2 and 15.6% were taking conventional synthetic and biological DMARDs, respectively. Further baseline characteristics, stratified by MTX use, are summarized in Table 1. MTX users were more often male, had a longer disease duration, and were less physically active. With regard to their GC use (history), those who used MTX had a higher cumulative GC dose, a longer cumulative duration of GC use, but were now using a lower dose than those without MTX use.

**Table 1:** Demographics and clinical features of the 188 patients with polymyalgia rheumatica and vasculitides stratified by MTX use

	Overall (*n* = 188)	No MTX use (*n* = 142)	MTX use (*n* = 44)	SMD
General information				
Age, years	68.10 (10.94)	67.81 (10.98)	69.02 (10.89)	0.111
Female sex, *n* (%)	124 (66.7)	98 (69.0)	26 (59.1)	0.208
BMI, score	26.57 (4.46)	26.31 (4.37)	27.42 (4.69)	0.245
Disease duration, years	2.82 [0.90, 8.56]	1.80 [0.83, 7.03]	4.67 [2.02, 9.49]	0.205
Health assessment questionnaire, score	0.25 [0.00, 1.00]	0.12 [0.00, 1.00]	0.25 [0.00, 0.88]	0.008
Regular physical exercise, *n* (%)	93 (49.5)	75 (52.8)	18 (40.9)	0.240
Alcohol consumption, *n* (%)				0.042
None	76 (41.8)	58 (41.7)	18 (41.9)	
Irregular/infrequent	79 (43.4)	60 (43.2)	19 (44.2)	
Occasional	23 (12.6)	18 (12.9)	5 (11.6)	
Frequent	4 (2.2)	3 (2.2)	1 (2.3)	
C-reactive protein levels, mg/l	4.80 [1.35, 11.00]	3.90 [1.22, 11.40]	5.40 [1.60, 9.60]	0.021
25-(OH)-Vitamin D in nmol/l	84.13 (25.50)	81.94 (27.23)	89.91 (19.32)	0.338
History of vertebral fractures	19 (10.2)	15 (10.6)	4 (9.1)	0.049
History of fractures due to inadequate trauma	62 (33.3)	45 (31.7)	17 (38.6)	0.146
Treatment and comedication				
Current GC dose, mg/d prednisone equivalent*	7.50 [4.00, 20.00]	10.00 [4.00, 28.75]	5.00 [2.15, 10.00]	0.385
Cumulative GC dose, mg	5963.50 [1825.00, 15,743.78]	5393.75 [1521.25, 12,423.62]	8946.88 [3315.00, 17,526.25]	0.056
Cumulative duration of GC use, years	2.08 [0.33, 6.93]	1.52 [0.22, 6.28]	3.13 [0.97, 7.98]	0.082
Proton pump inhibitor intake, *n* (%)	109 (58.6)	83 (58.5)	26 (59.1)	0.013
Data on MTX use				NA
Current MTX dose, mg/week			15 [10; 15]	
Cumulative MTX dose, g			1.46 [0; 5.37]	
Subcutaneous administration, *n* (%)^1^			17 (38.6)	
Current dose of s.c. users, mg/week			15 [12.5; 17.5]	
Oral administration, *n* (%)^1^			26 (59.1)	
Current dose of oral users, mg/week			11.25 [3.75; 18.75]	

Numbers are mean (standard deviation) or median (interquartile range) unless otherwise specified. *BMI* body mass index, *MTX* methotrexate, *SMD* standardized mean difference. ^1^*n* = 1 with missing data on administration *Includes patients without any glucocorticoid intake (as 0 mg/d)

### Comparing MTX users and non-users

**Fig. 1:**
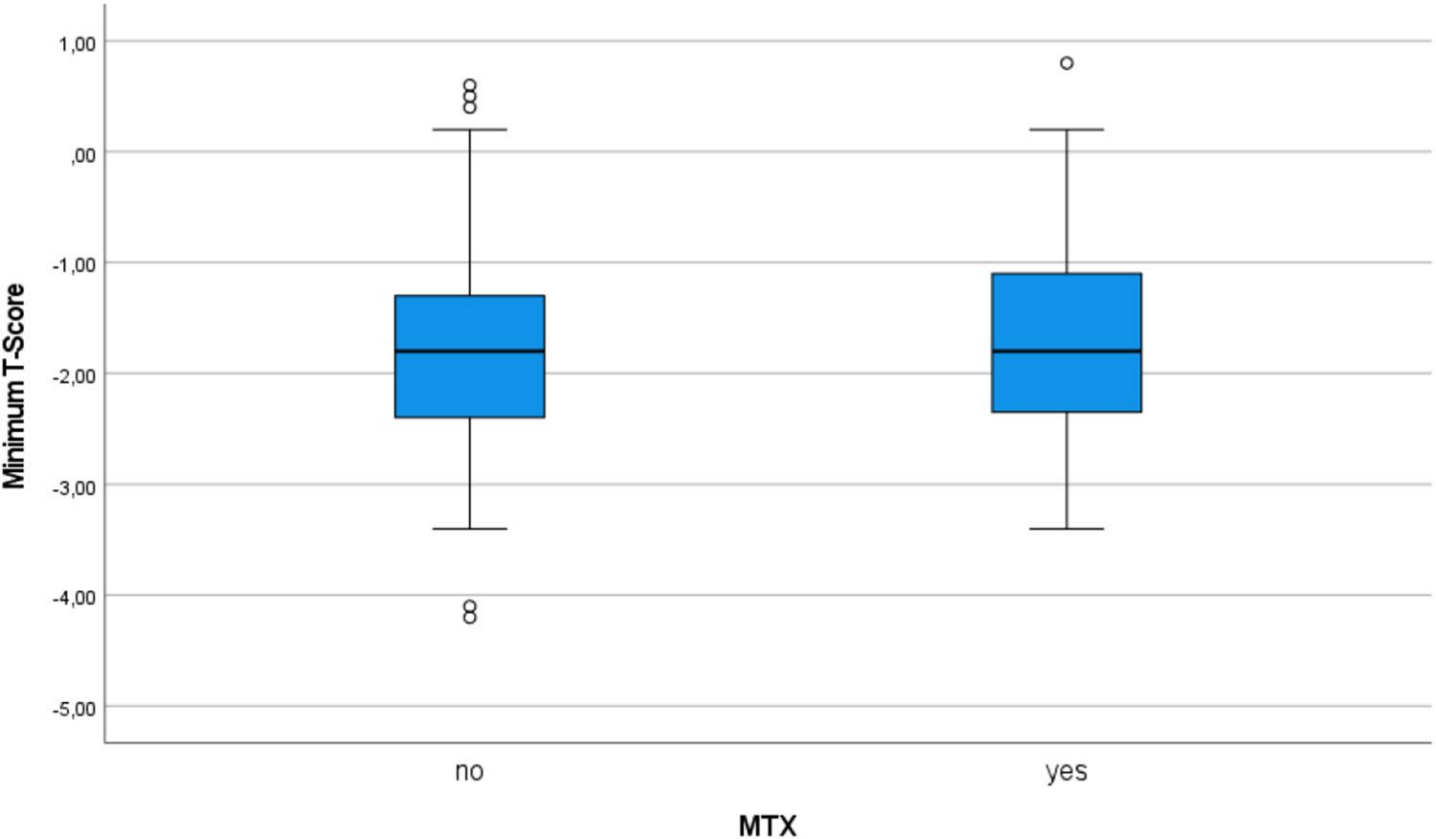
Box plot showing the minimum T-scores of MTX users and non-users (as observed). *MTX* methotrexate

In the unadjusted analysis, there was no statistically significant difference in minimum T-scores when comparing patients on MTX to those without MTX (difference -0.05; 95% confidence interval −0.36 to 0.26; *p* = 0.75; Fig. 1). Namely, patients taking MTX had a mean minimum T-score of −1.70 (± 0.86); those without MTX −1.75 (± 0.91). The results remained similar when comparing lumbar spine T-scores and total hip T-scores between MTX users and non-users in a sensitivity analysis. After adjustment for potential confounders in a multiple regression analysis, MTX users still had a similar bone density compared to non-users (slope −0.04; −0.34 to 0.25; *p* = 0.77); results remained similar after adjusting for serum C-reactive protein levels in a sensitivity analysis.

### Dose–response analysis

**Fig. 2:**
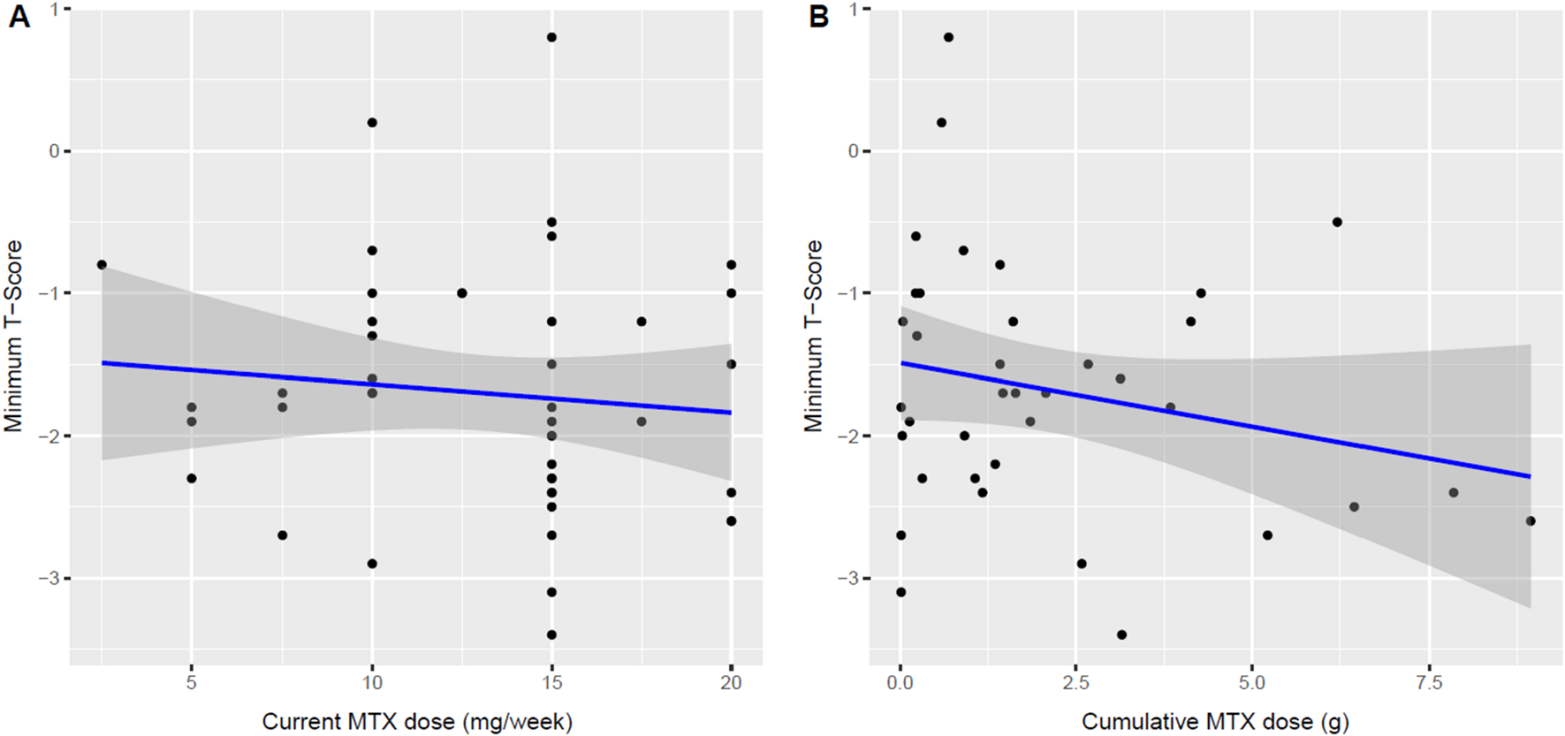
**A** and **B**. Scatterplots of minimum T-scores and *current* MTX dose **A** and *cumulative* MTX dose **B** within the group of MTX users (as observed). The regression lines are surrounded by their 95% confidence intervals. *MTX* methotrexate

The following analyses were restricted to MTX users. There was no link between current MTX dosage and BMD (slope −0.02; −0.08 to 0.04; *p* = 0.50; Fig. 2A) or cumulative dose (slope −0.03; −0.08 to 0.01; *p* = 0.17; Fig. 2B) and BMD in univariable analyses. Both current and cumulative dosages were also analyzed in a model adjusted for confounders, but the regression coefficients remained below the threshold for statistical significance (Table 2). Of note, the slopes were negative in both the unadjusted and adjusted analyses. After including serum C-reactive protein levels in a sensitivity analysis, the slope for cumulative dose was further reduced (indicating lower BMD in patients with higher cumulative MTX doses), but the *p *value remained above the threshold for statistical significance (slope −0.17; −0.35 to −0.00; *p* = 0.05). The results for current MTX dose were similar (data not shown).

**Table 2:** Multivariable analysis within MTX users

	Minimum T-score Reg. coefficient (95%CI)		*p *value
MTX current dosage (mg/week)	–0.02	(–0.14 to 0.09)	0.69
MTX cumulative dosage (g)	–0.12	(–0.28 to 0.05)	0.15

*MTX* methotrexate, *reg*. regression, *g* gram, *mg* milligram

## Discussion

In our cohort of patients with PMR, GCA and other forms of vasculitis, MTX use was not associated with BMD, and no significant dose–response relationship was seen within MTX users.

Patients with PMR and vasculitis have been shown to be at a greater risk of osteoporosis and fractures [2, 19]. Some causal factors for this increased risk have been proposed (e.g., GC intake and increased levels of inflammation), but the evidence base is fragile. Evidence comes mostly from observational studies whose findings have not been consistent. For example, Paskins et al. found a link between GC intake and the risk of fracture in a cohort of PMR and GCA patients, [2] but in a recent analysis of our Rh-GIOP cohort, we did not find an association between current or cumulative GC dose and BMD after adjustment for confounders such as age, sex, or BMI [3]. Consequently, we strive to identify further potential risk factors for impaired bone density in this cohort.

In the past, reports showed that (very) high doses of methotrexate, when used for cancer treatment, are linked to osteopenia and fractures [14]. These studies shaped the description and term MTX osteopathy as a rare separate disease entity, which is characterized by stress fractures of the lower extremities [20]. Henceforth, several studies addressing the role of MTX on bone have emerged, but no studies have investigated associations between MTX intake and bone health in patients with vasculitis. In the following, we will juxtapose our findings to studies in patients with other inflammatory rheumatic diseases.

In patients with inflammatory rheumatic diseases, MTX is commonly used in low doses (up to 25 mg/week). Studies specifically investigating low-dose MTX in the context of bone health are still few in number, often date back to the 1990s and early 2000s, and mainly assessed patients with rheumatoid arthritis (RA) or psoriatic arthritis (PsA). In summary, no clear evidence for a negative (or positive) impact of low-dose MTX on bone mineral density could be drawn from these investigations. This involves studies focusing on short-term effects as well as those investigating the impact of low-dose MTX over a long-term period:

When analyzing short-term effects of MTX on the bone, a large cross-sectional study with 731 female patients with RA compared MTX users (*n* = 246), who had received MTX for at least 6 months, to MTX “never users” (*n* = 485). BMD at the lumbar spine as well as at the femoral neck did not differ between the two groups after adjusting for age, menopausal status, BMI, Health Assessment Questionnaire score, and steroid use [21]. In another study, BMD of the radius/femoral neck/trochanter of patients with RA and PsA did not differ significantly between MTX-treated groups and controls [22]. A longitudinal study in patients with RA over 1 year did not show an adverse effect of MTX on bone turnover markers or histological features of bone formation from biopsies [23]. Although a univariate analysis found that MTX use at baseline was associated with a reduced BMD at the femoral neck, multivariate analysis and subgroup analysis of the subset of post‐menopausal women showed that reduced BMD associated with MTX was rather due to confounders such as disease activity.

In another longitudinal study (3 years duration) with 133 patients with RA, low-dose MTX use was not associated with changes in femoral neck or lumbar spine BMD in patients who were not treated with glucocorticoids. Changes were only noticed among those treated with concomitant prednisone ≥ 5 mg/day. Interestingly, these patients showed greater bone loss in the lumbar spine compared to patients with prednisone monotherapy [24]. It is, however, likely that treatment bias might have contributed to these results since active RA requires a more intense anti-rheumatic therapy. In a small study with an observation period of 2 years, MTX users and non-users lost bone significantly when compared to baseline, but did not differ from each other [25].

By juxtaposing our findings to studies in non-vasculitis patients such as RA and PsA, we need to point out that in the former, a clear link between inflammation and bone loss exists, locally in the form of bone erosions and systemically as osteoporosis. This may arguably not apply to the same extent to patients with vasculitis. Bone erosions are not pathognomonic for vasculitis, and the underlying inflammatory cascades differ from those of RA. Yet, in both, systemic inflammation seems to be a key driver of systemic bone loss. In untreated PMR patients, Barnes et al. showed bone turnover to be generally elevated [26]. Treatment with tocilizumab and GCs has been described to control bone turnover through inhibition of IL-6 [27]. Unfortunately, a homogenous assessment of disease activity in patients with various forms of vasculitis is less easy than in, e.g., RA. Using CRP or ESR as a global parameter for disease activity may also be misleading because CRP elevations in patients treated with DMARDs may have other causes. Also, tocilizumab inhibits the production of C-reactive protein.

A central limitation of this study is its cross-sectional nature from which no causal relationships can be assured. Also, the sample size of our study was rather small (too small for subgroup analyses), and our population was not homogeneous as different types of vasculitis (also including PMR) were studied. This was why we could not adjust for disease activity, as there are several different kinds of disease activity measures for each subtype of vasculitis. Instead, we performed a sensitivity analysis including serum C-reactive protein levels as a measure of inflammation and an indirect measure of disease activity. The results were not statistically significantly different, although a trend toward lower BMD with higher cumulative MTX doses was seen. Another limitation is that BMD is only a surrogate measure of the risk of fracture. Future analyses of the Rh-GIOP cohort will include this outcome provided that enough events occur. Finally, we did not analyze the impact of folic acid substitution which might have impacted BMD. A strength of the Rh-GIOP cohort is the large number of parameters, which allows for extensive adjustment for potential confounders such as GC intake. Another strength of our cohort is that the 25-OH-Vitamin D3 levels are mostly in the normal range and much higher than in the average normal population. Therefore, compared to other studies, we can largely exclude negative effects of hypovitaminosis D on the measured BMD.

We conclude that MTX use is not associated with bone health in our cohort of patients with PMR, GCA, and other forms of vasculitis.

## Data Availability

The datasets analysed in the current study may be made available from the corresponding and last author on reasonable request.
